# Consistent Evaluation Methods for Microfluidic Mixers

**DOI:** 10.3390/mi15111312

**Published:** 2024-10-29

**Authors:** Oliver Blaschke, Jonas Kluitmann, Jakob Elsner, Xie Xie, Klaus Stefan Drese

**Affiliations:** Institute for Sensor and Actuator Technology, Coburg University of Applied Sciences and Arts, Am Hofbräuhaus 1B, 96450 Coburg, Germany

**Keywords:** microfluidics, mixing measures, Dean flow mixer, simulation, computational fluid dynamics, FEM

## Abstract

The study presents a unifying methodology for characterizing micromixers, integrating both experimental and simulation techniques. Focusing on Dean mixer designs, it employs an optical evaluation for experiments and a modified Sobolev norm for simulations, yielding a unified dimensionless characteristic parameter for the whole mixer at a given Reynolds number. The results demonstrate consistent mixing performance trends across both methods for various operation points. This paper also proposes enhancements in the evaluation process to improve accuracy and reduce noise impact. This approach provides a valuable framework for optimizing micromixer designs, essential in advancing microfluidic technologies.

## 1. Introduction

The field of microfluidics has gained tremendous importance over the last decades, increasingly so with the proposal of the miniaturized total analysis systems concept in the early 1990s [1]. As a functional element in many point-of-care testing devices, microfluidic devices have become increasingly present in the lives of many people over the last few years.

One challenge in microfluidics is the mixing of fluids. Due to the small channel sizes, viscous forces dominate over inertial forces leading to a laminar flow [2]. Since mass transport occurs across the interface of streams, a large interfacial area is beneficial for mixing. In laminar flow conditions, the interface shape and area formed at the beginning of the flow do not change significantly, unless further measures are taken. The flow regime can be described by the dimensionless Reynolds number, Re. It allows for the comparison of flows of different media and environmental conditions, characterized by the density ρ and dynamic viscosity η of the fluid, the mean flow velocity *u*, and the hydrodynamic channel diameter $d_{h}$:

(1) $$Re=\frac{u\;\rho \;d_{h}}{\eta }$$

Laminar flows are characterized by low Re. At Re≈ 2000–2500, turbulent eddies can sustain themselves [3,4]. Depending on the inlet conditions, the critical Reynolds number is a range for the transition from laminar to turbulent flow conditions. The formation of turbulent flows in microchannels necessitates high flow rates at high-pressure losses and is usually not an option for the operation of microfluidic devices. In the case of microfluidic systems, however, the onset of turbulent flow characteristics has been observed at Reynolds numbers that are significantly below the critical Reynolds number previously cited. The most discussed and investigated reason for this is the increased significance of the wall roughness at shrinking channel sizes. As the hydraulic diameter falls, relative surface roughness rises to a point, where its effects cannot be neglected anymore for laminar flows. Besides wall roughness, entrance effects of developing flows, measurement uncertainties, and heating effects can also lead to deviations from expected flow behavior. These effects can become increasingly important at smaller scales, potentially leading to larger deviations that have to be accounted for [5,6,7,8].

Mixing can be characterized by the Peclet number, which describes if the mixing process is driven by advection or diffusion [9]. It can be defined as the product of the Reynolds number with the Schmidt number Sc, where the latter is the quotient of the kinematic viscosity ν and the diffusion coefficient *D*:

(2) $$Pe=Re\;Sc=Re\;\frac{\nu }{D}$$

For Pe>>1, advection-driven mixing is assumed, and for Pe≈1, the process is dominated by diffusion, which does not typically occur in micromixers and is therefore not of relevance to this topic [10,11].

While there are countless publications concerning micromixers, detailing the construction, operating principles, and characterization, no perfect micromixer has been presented to date. The suitability of a micromixer is determined by its application-specific requirements. These requirements can include factors like dead volume, integration space, mixing time, targeted mixing quality, range of flow rates, pressure drop, available material and surface quality, shear resistance of the sample, spatial resolution in the production process of the mixer, operational stability, and price. Due to the application specificity, the design and evaluation of micromixers is a perpetually ongoing process.

Within the last two decades, there has been a vast amount of publications addressing this issue. Different measures for mixing were developed and applied. One can differentiate between statistical measures like the mean or standard deviation of a concentration field [12,13,14] or stochastic measures that are based on probability distributions of tracers like Shannon Entropy [15]. Another approach is to use mathematical norms like a Sobolev norm to measure mixing, as investigated by Mathew et al. [16,17] or other groups [18,19,20,21,22,23,24]. Sobolev norms are capable of accounting for not only diffusive but also advective mixing, thereby enabling them to be employed at multiple scales and thus to provide a robust evaluation method. While the approach of using Sobolev norms appears to be advantageous, none of the literature has investigated whether these norms can be applied to actual experimental data and has focused solely on numerical experiments.

Computational fluid dynamics (CFD) is also often found in the literature as it can improve and accelerate the designing process of microfluidic mixing elements by giving insight into the fluid dynamics inside the microchannels resulting in a straightforward design process, in contrast to a trial and error approach [14,15,25,26,27,28].

For the in situ characterization of mixing processes, many methods are available, providing different advantages and drawbacks. The dye dilution method is the easiest process. A stream of a dissolved dye is mixed with a stream of solvent, and the distribution of the color intensity is evaluated. For a homogeneous dye distribution profile in the cross-section of the channel, complete mixing can be assumed. Since oftentimes just a projection of the channel cross-section is viewed, stacked or layered lamellae can pose a problem leading to erroneous evaluations of the mixing process [29]. This problem is overcome in color generation reactions such as the iron thiocyanate reaction. Two colorless compounds are mixed that yield a dye [29]. The absorption intensity rises with the amount of dye formed. It can be posited that the reaction is rapid and immediate upon mixing. The mean of the transmission over the projection of the channel can thus provide a representation of the mixing state, as it resembles the length of the boundary layer between the reactants. The absorbances for the pure and unmixed streams and mixed streams can be acquired and provide the boundaries of absorbances for unmixed, fully reacted, and hence, mixed states.

The aforementioned techniques provide only a projection of the channel cross-section subject to the Beer–Lambert law. Nevertheless, it is feasible to conceptualize the channel cross-section and ascertain the distribution of elements within the streams in the cross-section at specified channel positions. Techniques like confocal laser scanning microscopy offer the acquisition of the distribution of fluorophores over a channel cross-section [11,15]. Concurrent reactions such as the Villermaux–Dushman reaction are also frequently used for the characterization of mixers [30,31]. For the characterization of flow patterns, particle velocimetric methods can also be used.

The majority of studies available evaluate the mixing performance only at the outlet plane, therefore measuring solely the output of the mixing structure [14,26,27]. While giving information about a given structure, this single-point observation makes a legitimate characterization and thus a comparison between mixers impossible. There are some studies that take the length of the mixing element into account, measuring the mixing progression across its length, which results, depending on the used mixing measure, in either exponentially declining or logarithmic rising graphs [15,28,32,33]. This is a major improvement over the single-point evaluation, taking the progression of mixing and thus the character of the mixer into account.

A further area of investigation is how the aforementioned results could be used to enhance the analytical process. Therefore, experiments and simulations on a Dean flow micromixer at different operating points were conducted by varying the Reynolds number across a range of 0.5 to 400, and different mixing measures were compared as to which works best for capturing the mixing characteristic of the mixer across its length and also the residence time of the fluid within. The defined mixing measures consisting of statistical measures and a Sobolev norm with index −1/2 yielded exponentially declining curves, which were subsequently logarithmized to yield a linear representation. The slope of the linear curves was then acquired through fitting. The gradient was then de-dimensionized and plotted against the Reynolds number resulting in a characteristic map of the mixer. It was also shown that this approach works reasonably well for different mixer characterization approaches and also for CFD simulations where modifications to the calculation of the Sobolev norm were proposed to account for the throughput of the system. Some deviations, especially in the highest Reynolds number region, were found, which can most likely be addressed to manufacturing tolerances and sensor limitations, as well as numerical errors.

## 2. Materials and Methods

### 2.1. Subject of Investigation

In this study, a Dean mixer design was used to test the proposed approaches. This serpentine type of micromixer is well described in the literature and easy to manufacture, helping to investigate the expectations and limiting construction issues. The Dean mixer is known for its different secondary flow patterns depending on the Dean number and thus the Reynolds number. At the lower Dean number regime, two vortices, one located in the upper and another in the lower half of the channel cross-section, form due to inertia forces. At higher Dean numbers, an additional counter-rotating vortex pair appears, which increases the mixing performance significantly [34].

The operation principle of the Dean flow mixer is based on the generation of vortices that are normal to the flow direction. These vortices enlarge the interface area between the species and aid in accelerating the mixing process. The vortices appear upon surpassing thresholds in the Dean number, which characterizes flow in curvilinear channels. The Dean number, De, is defined with the Reynolds number, Re, the hydraulic diameter, $d_{h}$, and the mean radius of the channel curvature $r_{m}$ as

(3) $$De=Re\;\sqrt{\frac{d_{h}}{2r_{m}}}$$

An often-cited threshold for the formation of full vorticity in the flow is a Dean number of De=143 [34]. The necessity of a high De for efficient mixing requires high flow rates for a beneficial operation of a Dean flow mixer. An advantage of the Dean flow mixer is that its channels can be built planarly and without detached parts, thus facilitating the manufacture of this mixer [34].

Due to its simplicity and robust operation, the Dean flow mixer has found application for example in the synthesis of noble metal nanoparticles. In these syntheses, a fast mixing to a homogeneous dispersion is often important in the generation of seed particles. Without good and fast mixing, concentration gradients in the reaction are sustained, which leads to broad particle distributions and hence poor product definitions [35,36,37].

In this work, the geometry of the Dean flow mixer is characterized by six consecutive meander elements with $r_{m}=1.7$ mm, a channel width of 0.8 mm, and a channel height of 0.5 mm.

### 2.2. Experimental Setup

The Dean micromixer was built using polymer film layer lamination. The procedure was adapted from a rapid prototyping process for PMMA [38] and adjusted for the use of polycarbonate. The layers containing channels and access bores were outlined using Inkscape. The vector files were cut into polycarbonate sheets (LEXAN 8010; Dr. Dietrich Müller GmbH; Ahlhorn, Germany) using a VLS6.60 CO_2_ laser cutter (Universal Laser Systems, Inc.; Scottsdale, AZ, USA). The Layers were degrated and cleaned in isopropyl alcohol and water. The cleaned layers were sorted, aligned, assembled between microscopy slides, and clamped using paper foldback clips. The clamped assemblies were introduced into a preheated oven at 190 °C, after 3 min, a vacuum was applied for 3.5 min, and the assemblies were immediately removed from the oven.

**Figure 1:**
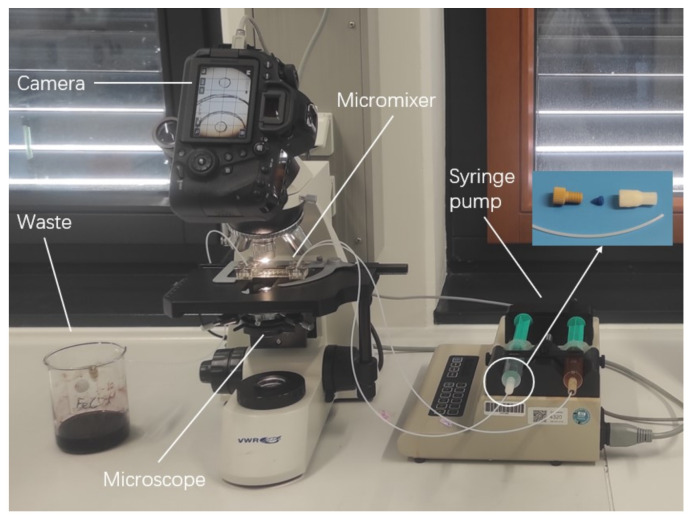
Experimental setup for conducting the color reaction and ink dilution experiments.

The mixing experiments were conducted by setting appropriate flow rates on a KDS 200 syringe pump (KD Scientific Inc.; Holliston, MA, USA). The reactants were loaded into plastic syringes of nominal sizes from 1 mL to 50 mL and were connected to the micromixers by pressing flanged tubings onto the access bores of the chips. Images were acquired on a VWR TR 500 microscope at 10×magnification (VWR International; Radnor, PA, USA), equipped with a Canon EOS 5D Mark IV Camera (Canon; Tokio; Japan) via an ocular port-to-EF-mount adapter. The experimental setup is illustrated in Figure 1. Exposure times were determined by flushing solvent, premixed reactants, and unmixed reactants through the illuminated channels and choosing conditions without over- or underexposure to the camera sensor. The exposure settings were fixed.

Chemicals were used as received without further purification. For the color generation reaction, iron(III) nitrate nonahydrate (≥96%, pure; Carl Roth GmbH + Co. KG; Karlsruhe, Germany) and sodium thiocyanate (ACS, 98.0% min.; Thermo Scientific Chemicals; Waltham, MA, USA) were dissolved in Ethanol (absolute ≥ 99.5%; VWR) to yield a 20 mM solution for FeNO_3_ and a 60 mM solution for NaSCN. For the dye dilution, Pelikan 4001 green ink (Pelikan Vertriebsgesellschaft GmbH & Co. KG; Hannover, Germany) was diluted 1:25 with deionized water prior to the experiment. Deionized water was used as diluent for the dye in the micromixer.

Confocal fluorescence microscopy images were acquired on a Leica Microsystems TCS SP8 microscope (Leica Microsystems GmbH; Wetzlar, Germany) with a 488 nm and a 552 nm excitation laser and appropriately set filter and detector configurations. Fluorescein disodium salt (extra concentrated; Carl Roth GmbH + Co. KG) and Rhodamine B (for microscopy; Carl Roth GmbH + Co. KG) at µM concentrations were used as fluorescent tracers.

### 2.3. Finite Element Simulations

**Figure 2:**
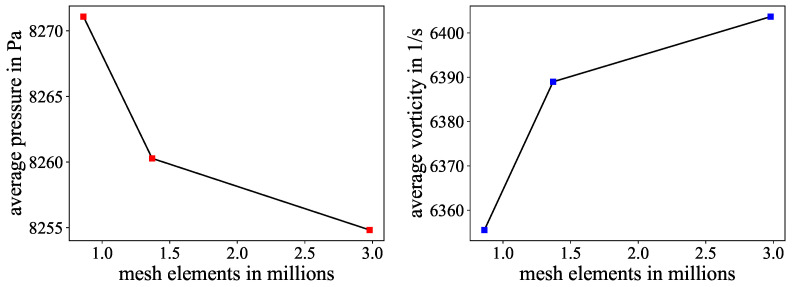
Simulation variables over different mesh refinement stages: (**Left**): Average pressure of the channel cross-section at the second meander. (**Right**): Average vorticity over the channel cross-section at the same position.

For comparison with the experimental data, finite element (FEM) simulations were conducted with COMSOL Multiphysics 6.1 software. The focus was on determining the extent to which simulations can reproduce the real mixer behavior of the chips. This is of relevance as computational experiments, with their easy access to cross-sectional data and insights into velocity profiles and fluid dynamics, can aid mixer development when linked with real experiments. The Dean mixer was modeled with the same dimensions and equivalent Reynolds numbers as in the experiments but neglecting numbers lower than 20. However, only the ink dilution experiment was simulated, as the color reaction required specific chemical parameters not fully detailed in the literature. The simulations used water properties at 20 degrees Celsius, matching the experimental conditions. The inflow boundary was split into two sections with different concentrations (1 mol/L and 0) to replicate the experimental setup. For the model, the symmetry of the structure was exploited by modeling only the lower half of the channel and applying a slip boundary condition on the top surface. The upper half information is acquired by mirroring the lower section in post processing. The model used hexagonal elements, which align sufficiently with the streamlines in comparison with tetrahedral elements. A mesh study was conducted for the use case of the highest Reynolds number, where the average pressure and average vorticity of the cross-section at the end of the first meander were compared for increasing mesh refinement stages, as shown in Figure 2.

**Figure 3:**
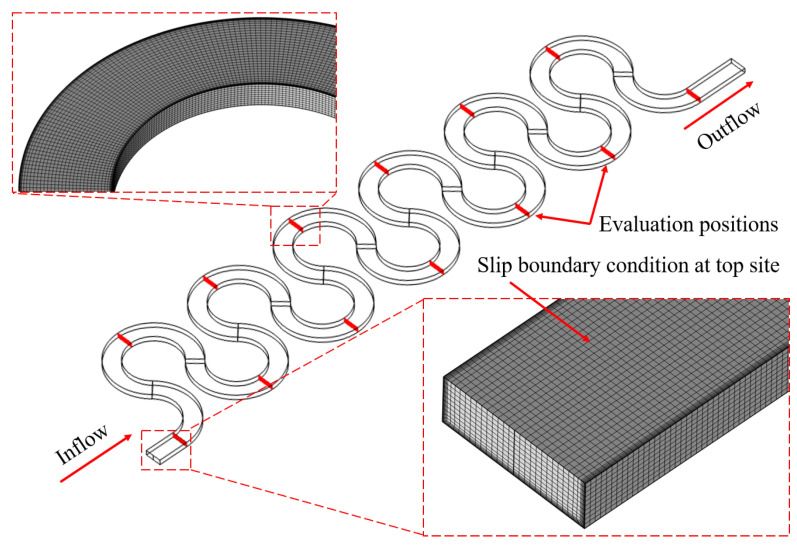
FEM simulation model of the Dean flow mixer with evaluation positions. Mesh element size is enlarged for visualization purposes.

From the graphs, a resolution of about 1.37 million mesh elements was chosen as a trade off between accuracy and computational costs. The definitive simulation domain and mesh can be seen in Figure 3.

During the simulations, the built-in stabilization schemes in COMSOL had to be utilized to prevent the simulations from diverging as the cell Peclet number, which is defined as

(4) $$Pe_{cell}=\frac{\bar{u}\;h}{2D}$$

with $\bar{u}$ as the advective velocity vector, *h* as the mesh element size, and *D* as the diffusion coefficient was much higher than a value of one, making the Galerkin method of the FEM unstable [39,40].

Although it was necessary to run the simulations, this approach brings some numerical diffusion into the system as a summand in the denominator of Equation (5), which acts on top of the molecular diffusion, thus having the potential to overestimate mixing through diffusion in general [41]. In COMSOL Multiphysics 6.1, there are two options for stabilization schemes. The first is an inconsistent method adding an isotropic diffusion to the overall system. The second one, which was also finally applied, utilizes streamline and crosswind diffusion, which only adds diffusion in areas where it is needed to lower the cell Peclet number, making the impact of numerical diffusion smaller as the first method [40,42,43].

To assess the amount of numerical diffusion introduced by the stabilization methods a diffusion-coefficient study for the range of the Reynolds numbers of this study was conducted. In the simulations, the molecular diffusion coefficient from the use case of the experiments with the ink dilution, which was $2.3\times 10^{-9}$ m^2^/s, was successively lowered to a value of $10^{-20}$ m^2^/s to approach zero diffusion. It was assumed that in the absence of molecular diffusion, the simulation system should exhibit only numerical diffusion (in addition to advection) in the results. Subsequently, the deviation in the dimensionless mixing time (the final mixing parameter of the study, which is discussed in detail in Section 2.5) between the simulations with molecular diffusion ($D=2.3\times 10^{-9}$ m^2^/s) and those with only numerical diffusion ($D=10^{-20}$ m^2^/s) was compared at varying Reynolds numbers. The rationale behind this approach is that if the deviation tends towards zero, it can be inferred that the simulation is predominantly dominated by numerical diffusion, rather than molecular diffusion. The deviation γ was calculated as

(5) $$\gamma =\frac{\tau_{mol}-\tau_{num}}{\tau_{mol}}\times 100\%$$

**Figure 4:**
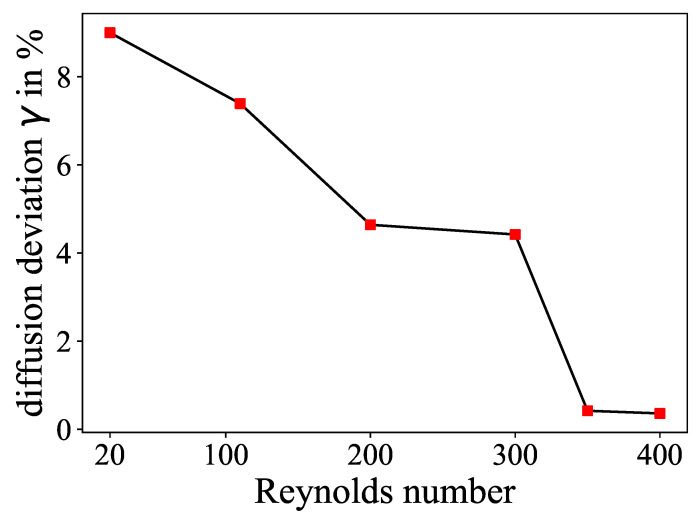
Deviation in percentage of the simulation results with molecular diffusion ($D\;=\;2.3\;\times \;10^{-9}$ m^2^/s) and only numerical diffusion (*D* set to $10^{-20}$ m^2^/s) across the occurring Reynolds numbers of this study.

As depicted in Figure 4, the deviation between the solutions with molecular ($\tau_{mol}$) and numerical diffusion ($\tau_{num}$) declines significantly for Reynolds numbers higher than 300. Although the overall deviations are relatively small, in the single-digit percentage range, this is to be expected as diffusion usually does not have a very large impact on advection-driven mixing problems. In this context, the increase in deviation with decreasing Reynolds number, and hence Peclet number, makes sense as diffusion becomes more important. However, the abrupt drop at the upper end of the Reynolds number range is striking. This may be an indication that the high degree of numerical error makes it questionable whether these simulation results resemble the diffusion in the physical experiments. The reason for the (nonlinear) increased amount of numerical diffusion may be related to the change in the secondary flow pattern of the Dean micro mixer at higher Dean numbers. Since the stabilization schemes used introduce numerical diffusion only where it is needed, the flow pattern could possibly contribute to the number of grid cells where the additional diffusion is introduced, thus affecting the total amount of added diffusion. Given that mixing in numerical approaches is commonly resolved using FEM simulations, the necessary calculations were performed, and the discrepancies between the simulations and the experiments are highlighted in Section 3.1.

### 2.4. Evaluation Scheme for Experiments

**Figure 5:**
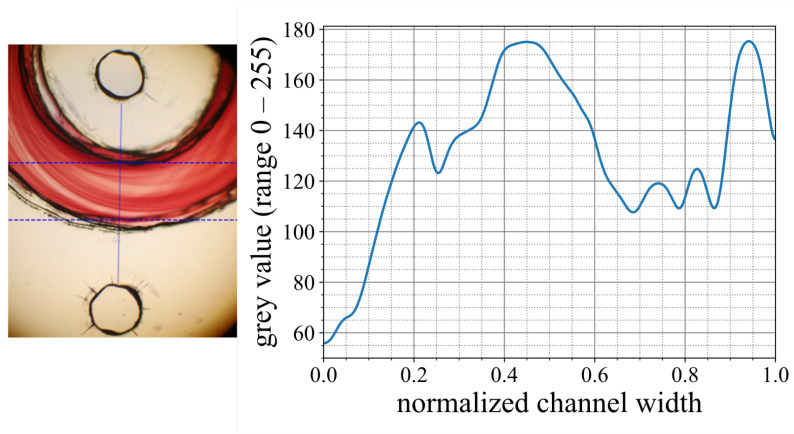
(**Left**): Microscope image of an evaluation section of the Dean flow micromixer. (**Right**): Gray values from the line measurement.

For the extraction of the gray values from the images of the experiments with the ink dilution and color reaction, a specially developed Python code was applied. According to Figure 5, the code separates the inner channel section from the channel walls and exports the gray value progression across the channel width.

The datasets of gray values were subjected to processing via the application of a Gaussian filter, with the objective of eliminating the high-frequency noise and thereby preserving the original shape of the gray value profiles. In a further analysis, several statistical measures were calculated for the dataset. These can be grouped depending on the type of experiment conducted. Lastly, the different metrics were evaluated across the length of the mixer or, alternatively, over the residual time of the fluid in the mixing structure.

Given that the color reaction experiment commenced with a channel that was nearly transparent and subsequently exhibited an increase in red value over the length of the mixer, it was imperative to utilize estimators that were indicative of the positional tendency, such as the mean or median. The Hodges–Lehmann estimator was also evaluated, as it is a more robust estimator for the median [44]. It is calculated from the median of the averages of pairwise sample points $X_{i}$ and $X_{j}$:

(6) $$H_{n}=\mathrm{median}\left(\frac{X_{i}+X_{j}}{2}:1\leq i\leq j\leq n\right)$$

**Figure 6:**
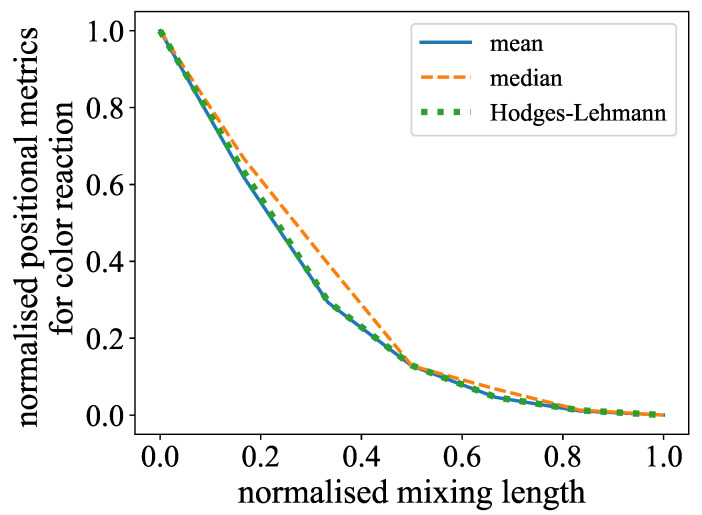
Comparison of mean, median, and Hodges–Lehmann estimator for the color reaction experiment at Re=170. Mean and Hodges–Lehmann estimators are very close. The median appears more unstable.

From these three measures, the mean and Hodges–Lehmann estimator were the most consistent. In particular, the median failed to give a reliable estimate in datasets where the middle region contained a high gradient in gray values, as they were then sensitive to an asymmetrical distribution, resulting in a fluctuation of the median, as can be seen in Figure 6. This phenomenon occurred especially in the low Reynolds number regimes, as the species were then mostly dominant in either the left or right channel half.

The ink dilution experiment required a measure that detected the change in the variance, as the mean of the ink–water solution was always the same across the channel cross-section. This resulted in the calculation of a measure of the dispersion of the data, which could be the standard deviation or a similar measure. The median absolute deviation (MAD) and the Shamos estimator are described both as robust alternatives to the classic standard deviation estimator and were therefore evaluated as well [44]. Meanwhile, the MAD is the median from the deviation of each sample point $X_{i}$ from the median of the dataset:

(7) $$\mathrm{MAD}=\mathrm{median}\left(|X_{i}-\mathrm{median}\left(X\right)|\right)$$

The Shamos estimator is defined as the median of the absolute deviations from all pairwise sample points $X_{i}$ and $X_{j}$:

(8) $$S_{n}=\mathrm{median}\left(\;|X_{i}-X_{j}|:1\leq i\leq j\leq n\right)$$

**Figure 7:**
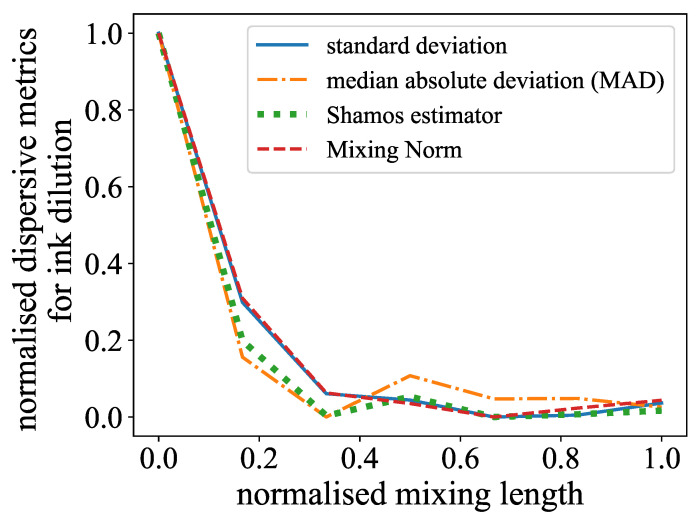
Comparison of standard deviation, MAD, Shamos estimator, and Mixing Norm for the ink dilution experiment at Re=170. Standard deviation and Mixing Norm give close results. Shamos estimator and especially MAD show large deviations.

In addition, a Sobolev norm of negative index was calculated as these types of norms can quantify the amount of dispersion and thus mixing [16]. The benefit of this method comes from the ability to not only measure mixing through advection through local variations in gray value distributions but also take diffusion into account by including information about the absolute values. This makes the so-called Mixing Norm a multiscale measure for mixing, and further details are presented in the following subsection, as this method is found to be superior for evaluating simulation results. As this approach works best if there is an unmixed initial field that gets mixed over time or length, it was only applied to the ink dilution experiments, resulting in a monotonous declining exponential representation (noise in the data beside). In regard to the color reaction, which commenced with a homogeneous, transparent channel, there was an increase in partial red lines, which subsequently became smoother. The representation of the curve is therefore a Gaussian form with a long tail. While this may be a reasonable assumption, it does present a challenge in the form of parameterization. This, in turn, makes the characterization process more difficult. Concerning the evaluation of the ink-based experiments, it was also determined that the Mixing Norm had very similar behavior to the standard deviation. The MAD and Shamos estimator performed worse, as they rely again on the median, which is not ideal for this use case, as stated before and seen in Figure 7.

**Figure 8:**
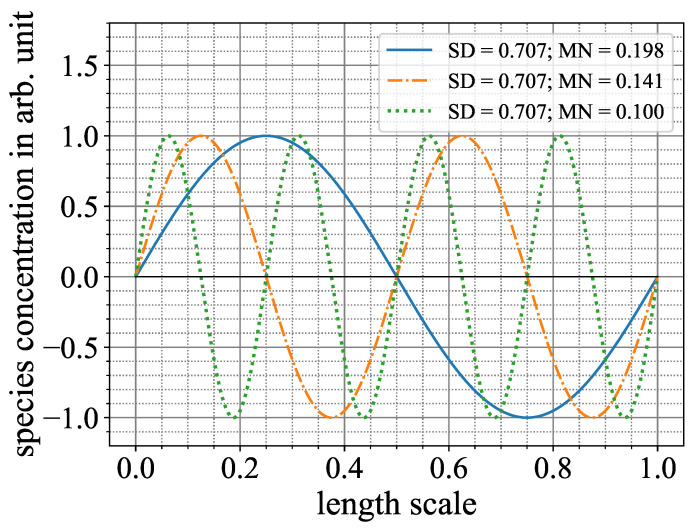
Comparison of standard deviation (SD) and Mixing Norm (MN) on a sinusoidal mixing map. While the standard deviation remains constant at different levels of advective mixing, the Mixing Norm tends to zero tracking the mixing process.

While the Mixing Norm comes at a slightly higher numerical cost, it can provide a meaningful result even in use cases where no or only a small amount of diffusion takes place, for example, the mixing of proteins. The reason why the Mixing Norm and the standard deviation perform very similarly here is that, despite the latter one only being able to measure the dispersion (the change in amplitude of the gray values) directly, it measures the advection in an indirect manner, as a high advection also accelerates the diffusion process. In an example in Figure 8, it can be seen that in the absence of diffusion, the standard deviation remains constant, even when the number of lamellae increases. In contrast, the Mixing Norm performs meaningfully and tends to zero.

The aforementioned screening led to mean and Mixing Norm curves over the mixer length, which represented an exponential decline. The length of mixing was then transformed into a residence time by taking the mean flow velocity of the experiments into account. A logarithmic transformation led to the linearization of the exponential curves and made the fitting of a linear model possible. The absolute value of the gradient of these linear models α has a unit of 1/s, which has to be de-dimensionized before it can be used for comparison. Therefore, a dimensionless mixing time was introduced by multiplying α with the squared hydrodynamic diameter $d_{h}$ and dividing by the kinematic viscosity ν:

(9) $$\tau =\frac{\alpha \;d_{h}^{2}}{\nu }$$

This approach is valid for advection-dominated mixing processes where the viscosity and the resulting viscous forces are the most important. In short, processes where Pe>>1 applies. On the other hand, for diffusion-driven mixing processes, one has to replace the kinematic viscosity ν with the diffusion coefficient *D* in Equation (9), as this is therefore the main source of mixing. After this adjustment, the resulting dimensionless mixing time was plotted against the Reynolds number of the corresponding experiment resulting in a map of the mixer performance for various points of operation. The associated graphs are depicted in Section 3.1.

### 2.5. Evaluation Scheme for Simulations

The evaluation of simulation data required a different approach from the experimental data. In three-dimensional simulations, the information is usually represented in a two-dimensional cross-section of the channel, focusing on the change in the concentration profile.

A crucial step in the evaluation process involved weighting the concentration profile with the local mass flow, similar to calculating the Nusselt number in thermodynamics, where the calculation of the average bulk temperature is essential [45]. This was necessary to account for the throughput of mixed or unmixed fluid in the system. As the density of the fluid and the cross-sectional area were at any points constant, the concentration profile *c* was multiplied by the normalized velocity profile of the main flow direction $u_{x}$ instead of the actual mass flow. Further, the mean concentration had to be subtracted to center the field around zero.

(10) $$c_{weight}=\left(c-c_{mean}\right)\cdot \frac{u_{x}}{max(u_{x})}$$

In this case, the Mixing Norm is again a useful tool to calculate the amount of mixing. The Mixing Norm, mathematically a Sobolev norm of negative index, can be described as an extension of the L2 norm incorporating information about not only the size of a function but also the size of its derivatives, allowing statements about the function’s decay. The general form of a Sobolev norm for a function that is represented as a Fourier series is given as

(11) $${∥f∥}_{H^{s}}={\left(\sum_{k=-\infty }^{\infty }{\left(1+{\left(2\pi ||k||\right)}^{2}\right)}^{s}{\left|c_{k}\right|}^{2}\right)}^{1/2}$$

Here, $c_{k}$ are the Fourier coefficients with the corresponding wave numbers *k* that represent the weighted concentration profile. The exponent *s* is the index of the Sobolev norm, which is −1/2 for the Mixing Norm, indicating the evaluation of the half derivation in the negative Sobolev space. In theory, this smoothing makes it possible to evaluate functions that are usually not differentiable. The use of a 2D Fast Fourier Transform algorithm allowed for easy computation of the underlying function of numerical data. In the analysis, the Mixing Norm values were plotted against the residence time, linearized through logarithmic transformation, and de-dimensioned, which is the same procedure as with the experimental data according to Equation (9) to determine the dimensionless mixing time. The absolute values of the latter were then correlated with the Reynolds number and compared with the experimental results.

While this method provided the most information, it was possible to investigate the effect of averaging across the channel depth similar to the Beer–Lambert law in the experiments where the evaluation was only from the top of the microfluidic chip. Here, a mean value for each column of the two-dimensional data was calculated, reducing the two-dimensional image to a one-dimensional line interpretation similar to the experimental gray value data. From here, a 1D Mixing Norm was calculated just like in the ink dilution experiment. The subsequent section presents a comparison of the one- and two-dimensional evaluation schemes for the simulation and the experiments. The confocal image analysis at the end of the section demonstrates that it is possible to apply the proposed methodology to even advanced measuring schemes.

## 3. Results

### 3.1. Comparative Mixer Map

**Figure 9:**
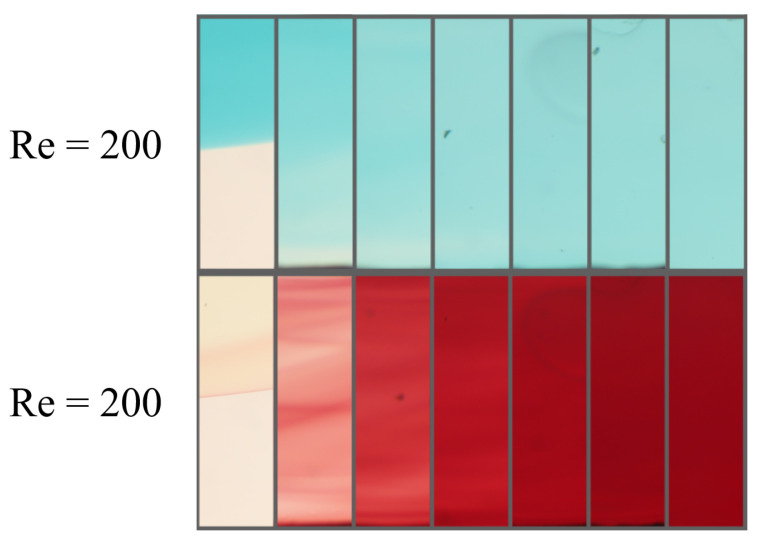
Consecutive image sections of the evaluation positions for the ink dilution experiment (**top**) and the color reaction experiment (**bottom**). Both at a Reynolds number of 200.

In this section, the different experimental and simulation evaluation approaches are presented and compared. Starting with the line evaluation experiments, both types are displayed in Figure 9. The consecutive sections display the mixing progress for the ink dilution and color reaction experiments for the operating point at a Reynolds number of 200.

**Figure 10:**
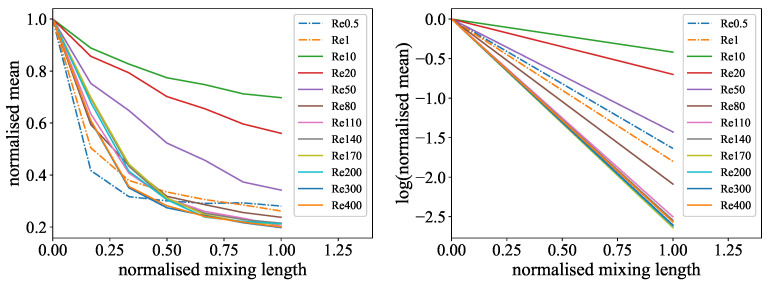
Length-based evaluation of the color reaction experiment: (**Left**): Normalized mean values for different Reynolds numbers over the normalized mixing length. A higher exponential decline resembles a shorter mixing length. (**Right**): Linear fit representation of the logarithmized exponential mean curves for different Reynolds numbers over the normalized mixing length. A higher slope indicates a shorter mixing length.

**Figure 11:**
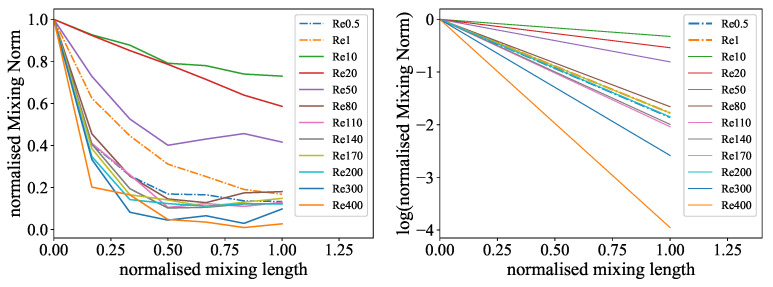
Length-based evaluation of the ink dilution experiment: (**Left**): Normalized Mixing Norm values for different Reynolds numbers over the normalized mixing length. A higher exponential decline resembles a shorter mixing length. (**Right**): Linear fit representation of the logarithmized exponential Mixing Norm curves for different Reynolds numbers over the normalized mixing length. A higher slope indicates a shorter mixing length.

The resulting mixing measures, the mean and the Mixing Norm, from the evaluation of the experiments are plotted against the normalized mixing length, as well as the linear fitting curves for the logarithmic representation in Figure 10 and Figure 11. On both of the left sides, it is noticeable that for all experiments, there is an exponential decline in the mixing measure indicating the progress of mixing. The linear fitting curves in the same figures are noticeable on the right side, where all graphs above a Reynolds number of 140 are very close together and the typical sorting from the lowest Reynolds number to the highest seems lost. Furthermore, it can be observed that the curves for the experiments with low Reynolds numbers (0.5 and 1) do not align with the overall trend, where higher Reynolds numbers are associated with higher slopes. This is because at this flow regime, the mixing process is considerably driven by diffusion, indicating that this evaluation technique is not fully appropriate for this type of experiment.

**Figure 12:**
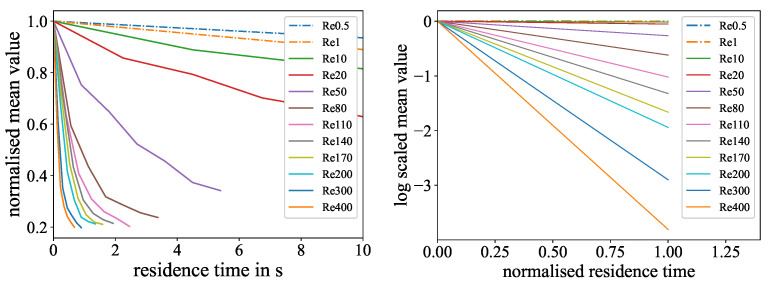
Time-based evaluation of the color reaction experiment: (**Left**): Normalized mean values for different Reynolds numbers over the residence time in the mixer. A higher exponential decline resembles faster mixing. (**Right**): Linear fit representation of the logarithmized exponential mean curves for different Reynolds numbers over the normalized residence time. A higher slope indicates faster mixing.

**Figure 13:**
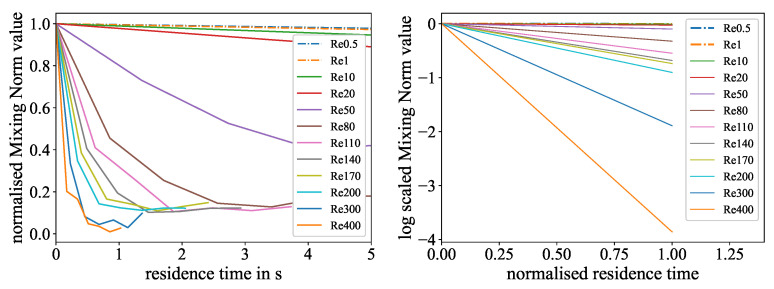
Time-based evaluation of the ink dilution experiment: (**Left**): Normalized Mixing Norm values for different Reynolds numbers over the residence time in the mixer. A higher exponential decline resembles faster mixing. (**Right**): Linear fit representation of the logarithmized exponential Mixing Norm curves for different Reynolds numbers over the normalized residence time. A higher slope indicates faster mixing.

To address this issue, a time-based evaluation was conducted. Here, the mixing length was transformed into a residence time with the main flow velocity obtained from the Reynolds numbers and viscosities. In this representation, the curves are consistent, even for the lowest Reynolds numbers, which are visualized in Figure 12 and Figure 13.

**Figure 14:**
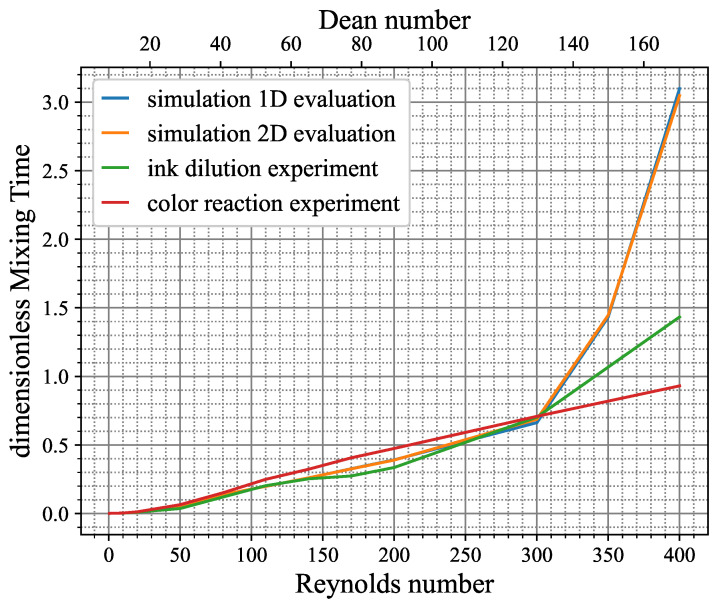
Mixing map for the Dean mixer showing mixing performance at different Reynolds numbers. Comparison between experiments and simulations.

In the subsequent phase, the fitting parameters derived from the linearized curves of the time-based evaluation were de-dimensionized by the approach in Equation (9) and plotted against the Reynolds number as the dimensionless mixing time. The very same approach was conducted for the results achieved by the simulation technique with the one- and two-dimensional evaluation scheme. The overall results are presented in Figure 14.

**Figure 15:**
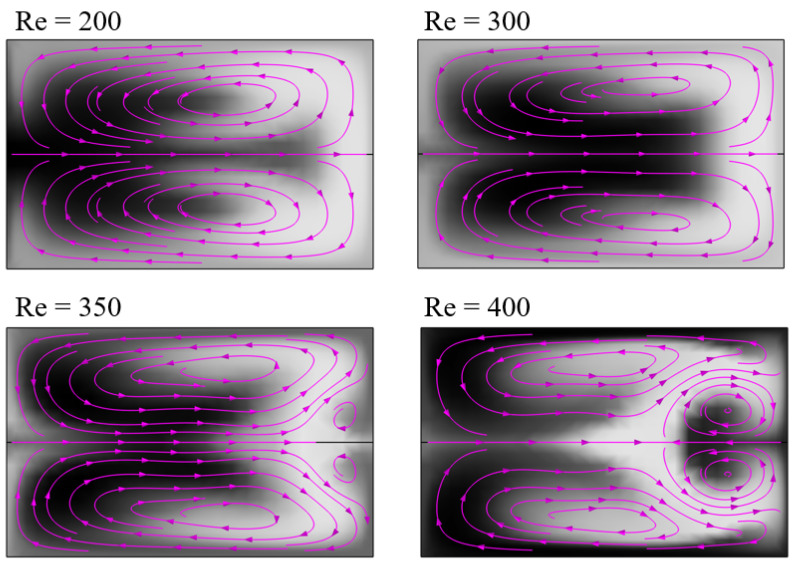
Secondary flow pattern of same evaluation position from Dean mixer simulations at different Reynolds numbers. Concentration profile as grayscale map, with black equal to high concentration and white low.

In the simulation, the dimensionless mixing time showed an exponential rise with increasing Reynolds number. In fact, there were two different exponential slopes, with Re=300 as turning point, with a sharp rise at higher Reynolds numbers. This correlates with the formation of additional vortex pairs in the flow pattern at higher Reynolds numbers (and thus Dean numbers), as depicted in Figure 15. A small deviation was observed comparing the one-dimensional and two-dimensional evaluation approach of the simulation data, but the difference is negligible. This can be interpreted as the top-view evaluation approach of the experiments being valid, as the averaging over the channel depth seems to only have a small effect, at least in the conducted study.

The curves from the experiments both showed a good agreement with the simulation data till the Reynolds number of 300, with the ink dilution being closer to the simulation as the color reaction. It has to be stated that the data point of Re=350 is only available in the simulation dataset and is missing in the experiments, making Re=400 the only data point above Re=300 for these curves. While the ink dilution graph showed a steeper increase above Re=300, similar to the simulation data, the final value at Re=400 had a large deviation from the simulation. This conspicuousness supports the thesis in the preceding section that a high extent of numerical diffusion leads to an overestimation of the mixing performance in the simulation. It is noteworthy that the overshoot phenomenon occurred exclusively at the highest Reynolds numbers, where the maximum level of numerical diffusion was introduced into the system, as was already found in Figure 4 in Section 2.3. This phenomenon appeared to be caused by the improved advective mixing, which is a consequence of a significant alteration in the secondary flow profile. This resulted in a higher number of grid cells affected by numerical diffusion to compensate for the instability problem of the Galerkin method of the FEM, as described in Section 2.3, and thus led to an increased total amount of numerical error. As a consequence, the simulation solutions for the Reynolds numbers 350 and 400 are most likely invalid, as the diffusion study from Figure 4 suggests. An attempt was made to reduce the numerical diffusion for the simulation with Re=400 to achieve valid results. It was found that it was not feasible to achieve convergence for this case without utilizing the stabilization schemes that introduce the numerical diffusion embedded within the COMSOL software. As illustrated in Equation (5), the only viable method for reducing the numerical diffusion in FEM is to reduce the grid element size considerably, which is not a viable option in a practical sense due to numerical costs.

The color reaction graph exhibited similar but higher values than the others until Re=300. However, above this point, no discernible change in the slope of the graph could be determined, as it appeared to increase linearly. This is notable given that all the other graphs demonstrated a significant increase.

The general deviation at Re=400 in the experimental data can be addressed not only by the numerical diffusion of the simulation method but also by the manufacturing quality of the mixing channel, preventing the optimal vortex formation. Further, the linear course of the color reaction indicates an issue with this type of experiment itself, as the further progress in mixing could not be resolved.

### 3.2. Confocal Microscope Imaging

**Figure 16:**
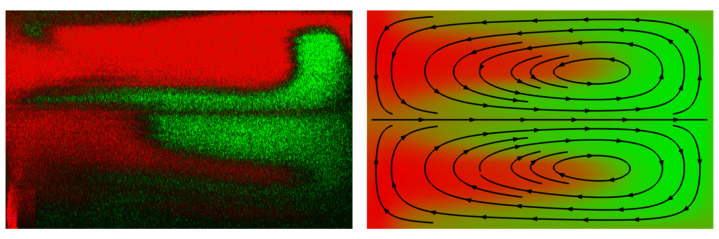
Confocal microscope image (**left**) and simulation cross-section of the concentration field (**right**) for the Dean mixer at Re=80 on the end of the fourth meander element. In the simulation representation, arrows indicate the direction of velocity streamlines. The colors red and green represent the species concentrations.

For a more in-depth analysis of the manufactured Dean mixer chip, images of the channel cross-sections were obtained through confocal laser scanning microscopy. Two distinct positions within the microchannel were imaged, with two different Reynolds numbers. A comparison of the results revealed similarities to the corresponding FEM simulation, as illustrated in Figure 16. The center part of the distribution especially closely resembled the simulation, and even the recirculation at the bottom of the channel was visible. Conversely, there were also areas where the concentration profile of the experiments did not align as expected, for instance, the recirculation at the upper portion of the channel displayed in Figure 16. It was found that the polymer layers from which the chips were manufactured were slightly misaligned, leading to steps in the channel walls. Due to the diverging beam of the laser cutter, the channel walls were also slightly angled. These limitations were a consequence of the manufacturing method selected, resulting in discrepancies between the designed micromixer and the final product. For mixing quantification, the Mixing Norm for the images was calculated and compared at the same channel cross-section for the Reynolds numbers 10 and 80. Given that the Mixing Norm value was 0.127 for Re=10, which is higher than the value of 0.081 for Re=80 at the end of the fourth meander, it can be posited that the proposed evaluation scheme can be also adapted to this type of experiment. However, further experiments must be conducted to provide substantiation for this assertion.

## 4. Discussion

This study introduces a method for evaluating micro mixer performance in both experimental and simulation setups, assessing mixing not just at the outlet but throughout the entire mixer geometry for a more comprehensive approach. The experiment relies on an optical evaluation scheme, tracking the change in pixel gray values and derivation of a single characteristic dimensionless fitting parameter for the complete mixer at a given Reynolds number, whereas the simulations evaluate the two-dimensional cross-sections along the mixer with a Sobolev norm and also lead to the same kind of parameter.

It has been shown that there is a similar trend for the mixing performance in both experiments and simulations over a wide range of operation points of the mixer, demonstrating the potential of this method for understanding and optimization of mixing structures. It was also shown by simulation that the optical top view evaluation and line interpretation provides a sufficient estimate of the mixing in the actual cross-section, at least for the conducted experiments.

It was observed that, for the highest Reynolds number in this study, the evaluated dimensionless mixing time showed a significant discrepancy between simulation and experiments. The ink dilution experiment exhibited at least a similar trend for this point, with an increasing slope of the parameter curve indicating the formation of additional vortices in the secondary flow pattern in the cross-section of the Dean mixer. However, the color reaction remained at a constant rate.

In order to achieve a unified outcome, it is recommended that the aforementioned discrepancies be addressed by undertaking a process of critical analysis to identify areas of improvement. Considering the experiments, the manufacturing technology with the limited resolution of a stepper motor positioned CO_2_ laser cutter and the possibility of material creepage of ductile materials like polycarbonate can possibly negatively impact the dimensional accuracy and hence the comparability between simulative and experimental results. While the impact of bubbles was reduced by using ethanol as a solvent in the thiocyanate reaction to flush bubbles out of the reactor, and the syringes and connections were bubble-free, a possible improvement could be the evaluation of a set of lines per evaluation position and averaging the local results to reduce the sensitivity to interferences like local microbubbles and particles for noise reduction.

In the simulation procedure, it is recommended to use other techniques than FEM as the instability problem, and thus, introduced numerical diffusion seems inevitable. Alternatively, another common approach to CFD (Computational Fluid Dynamics), the finite volume method (FVM), can be used. Nevertheless, FVM could also experience issues with numerical diffusion, as the numerical approximation of the advection term of the advection–diffusion equation leads to errors, especially if the mesh grid surfaces are not orthogonal to the flow direction [41,46,47]. Another usual approach is the usage of particle tracing methods where particles are guided by streamlines, and Poincaré maps can be evaluated [48]. Drawbacks are the lack of mass conservation and the incapability to model diffusion effects as the particles do not interact with each other. Full Lagrangian simulation methods like Smoothed Particle Hydrodynamics are a promising approach, as they are not exposed to numerical diffusion and satisfy mass conservation [49]. As these methods are inherently solved transient rather than stationary, higher numerical costs and calculation times are to be considered.

The next phase in the evaluation involves correlating mixing performance with the energy consumption required for mixing. This is crucial for assessing efficiency and the comparison of different mixers. Energy consumption, indicated by the pressure drop across the mixer, can also be recorded in experiments and simulations and will be a focus in future work, as well as the proposed optimization methods for both experiments and simulations.

## Data Availability

The Dataset is available on request from the authors.

## Abbreviations

The following abbreviations are used in this manuscript:

CFD	Computational Fluid Dynamics
FEM	Finite Element Method
FVM	Finite Volume Method
MAD	Median Average Deviation
